# Illinois State Dental Society, 1897

**Published:** 1897-09

**Authors:** 


### AMERICAN JOURNAL

—OF—

### DENTAL SCIENCE.

VOL. XXXI. BALTIMORE, SEPTEMBER, 1897. No. 5.

ARTICLE I.

### ILLINOIS STATE DENTAL SOCIETY, 1897.

### DISCUSSION ON DR. WASSALL'S PAPER ON "PYORRHEA ALVEOLARIS."

Dr. A. W. Harlan, Chicago: This paper of Dr. Wassall's deals with a subject that we all have more or less acquaintance with, and I do not expect in opening the discussion to deal with all of the divisions of the subject that he has made. In alluding to the definition, Dr. Wassall states that Wedl had previously described what we know as pyorrhea alveolaris. He also named it pyorrhea alveolaris. This book was published about thirty years ago and was translated into English as early as 1872, so that the name pyorrhea alveolaris had become familiar to a good many English-speaking peeple through reading that translation. Previous to its appearance in English, Dr. J. H. P. Brown, of Augusta, Georgia, had described this disease without being acquainted with Wedl's classification or denomination, and it was pretty well known from that description, although no name was given to it. As early as 1877 the late Dr. Rehwinkel, of Chillicothe,

Ohio, read a paper before the American Dental Association which was devoted to a consideration of what was termed pyorrhea alveolaris, and later, as Dr. Wassall has said, Dr. Black described one phase of it, one division of it, as phagedenic pericementitis. I think probably even now to a mixed audience the distinction between what is known as pyorrhea alveolaris and phagedenic pericementitis is not clearly understood. I should say, from a somewhat close observation of a large number of cases, that we find in connection with loosening of teeth not only a detachment of the peridental membrane and wasting of the alveolar process, but another condition present, the development of pockets. In the first case, we have the loosening of the teeth, the wasting of the alveolar process, and a gradual evulsion of the teeth without any deposit whatever, no deposit being found at any time, the teeth coming out clean and divested of the peridental membrane. In the other phase we find that there are pockets, and concomitant with them, or shortly following the development of these pockets, we have deposits that have been named serumal or sanguinary deposits, (Ingersoll). Those deposits are granular or smooth, in sheets or islands, and they may or may not cover the whole side of the root of the tooth as far as the pocket extends along its side. In most of these cases we also have the breaking down of the alveolar process, not symmetrical, but projecting, so that the alveolar process may surround the lingual surface of the tooth and the root, and the alveolar process will be broken down on the labial surface. Now, we have a totally dissimilar condition in that case from the one in which there is a uniform wasting of the socket, with loosening of the tooth and destruction of the peridental membrane.

In all of the cases of that kind that I have ever seen or examined or observed, I have never yet found one where there was a deposit on the side of the root or near the pockets or covering the apex of the root but what there was some method of reaching it from the gingival margin. I have heard speakers say in conventions before now that these nodules or islands have been found without there being any detachment of the gum at the gingival margin. I have never seen one of these cases. I have seen them, however, when the pulp of the tooth had previously been destroyed, and the root of the tooth had been imperfectly filled, and then I have seen these deposits after the teeth were extracted, but there was no pericemental detachment. If such a condition of things exists whereby a deposit or nodule of calcific matter could be laid on the side of the cementum without any opening leading from the gingival margin, why do we only find such things on one tooth? Why would we not find it on two or three? I can not answer that question. I do not know. It seems to me that in all of these cases the presence of micro-organisms is absolutely necessary, and that we are concerned in the first place with the loosening of teeth and the symmetrical destruction of the alveolar processes with an anærobic micro-organism described by Pasteur as early as 1870, I think; and in the formation of deposits on the roots of teeth, in pockets or pouches, that we are concerned with the anærobic and the facultative micro-organisms, the anærobic micro-organisms being able to subsist and destroy tissues without the absolute presence of oxygen, and the facultative organism working as a pathogenic micro-organism that subsists in the presence of or being deprived of oxygen. These minute deposits and granules and sheets, I should say, are probably, without knowing anything more definite, the result of the destruction of the hard tissues surrounding the root and the deposition at that point, because the roots which contain the products of these organisms are not exposed to any of the fluids sufficient to wash them away, and so in these deposits we find there remain the old and broken-down facultative and anærobic organisms. This is very clearly shown by some of the work of McFarland in his book on "Pathogenic

Bacteria," and it is further shown in the work of Park in the "American Text book of Surgery," on the "Deposits on Long and Flat Bones," but always where there is an open sinus or an opening to the external world. By analogy, if the same process causing the destruction of a portion of the periosteum in bones and the destruction of a portion of the bone itself, or a destruction of the nutritive material which is carried to the point which is loaded with inorganic materials, probably we have reason to believe that that may be the method of the deposition of these deposits independently of the uric acid, rheumatic, gouty, or other diathesis.

Dr. Wassall in his paper said that he did not quite wholly subscribe to the theory of Pierce and Kirk with reference to all of these cases being attributed to the uric acid diathesis. It has been my good fortune to have had under my care a few people who were strict vegetarians, and it is not generally believed that vegetarians are subjects of the uric acid diathesis, but they had loosening of the teeth and so-called or actual pyorrhea alveolaris. It does not seem to me that the theory of the universal production of nodular deposits on the roots of teeth is wholly accounted for by the theory advanced by Pierre and Kirk, as the whole human race can hardly be said to possess a universally rheumatic or uric acid diathesis. I know we are sailing in deep water when we are talking about the deposition of these islands and granules and nodules on the roots of teeth, but we have them present, and if we can prevent injury to the gingival margin, the gingival attachment to the necks of teeth might perhaps solve that question But we have an actual loosening of the teeth, and we have to meet that condition of things.

The method of administering medicines internally for for the cure of what we term pyorrhea alveolaris without surgical treatment or removal of the deposits is an absolute failure. The deposits must be removed. The administration of various forms of lithia and lithiates and gaseous waters for the relief of the loosening of teeth, where there are no deposits, does not result in a cure. It has been absolutely necessary to fix these teeth with splints or bands or some method of holding them together by running bars across the ends or wires, or something of that sort; and the application of local remedial agents.

In the consideration of the causes of pyorrhea alveolaris, in a paper read by Dr. Rhein, of New York, a year and a half ago, he attributes everything practically to malnutrition; but you and I know, and nearly everybody knows, that some of the most robust and active and outdoor people have loosening of the teeth. They may have nasal obstructions; they may have diseases of the mucous membrane itself which will cause them to breath through the mouth. Now, in those cases we have true pyorrhea alveolaris—that is to say, so distinguished from phagedenic pericementitis, with the destruction of the alveolar process, of the peridental membrane, loosening of the teeth, distortion of the teeth, etc.; without any uric acid diathesis or complications. I should say, then, pyorrhea alveolaris with a half-dozen other names, such as infectious alveolitis (Witzel), and loculosis alveolaris (Farrar), symptomatic alveolar arthritis (Magitot and Malassez), subscribed to by Gallipe, of Paris, who has paid a great deal of attention to this subject, with all of these various aspects, we have a great variety of complications, all of which need more or less different terms, and so far as I am concerned, I have never been able to follow a cast-iron rule in the treatment of these cases.

There are two points in connection with pyorrhea alveolaris that I would like to emphasize, as spoken of in Dr. Wassall's paper. They are these: One is this, that the teeth must be fixed in their sockets, held as nearly firm as it is possible to hold them, permitting of a minimum movement. The other point is that the deposits on the roots of teeth in pockets and pouches must be removed. When that is done, cleanliness, the frequent use of various agents in the mouth to destroy any infection, and the sufficiently frequent use of systematic medication to the pouches or pockets is all we can do to effect a cure.

With reference to recurrence. It seems to me there are a number of cases that are cured, and they do not recur. This depends entirely on the local cleanliness and care, with a reasonable amount of inspection by the dentist.

Dr. R. N. Lawrence, Lincoln: I have no criticisms whatever to make on Dr. Wassall's paper. He has given us a clear, concise resume of the outlines of this disease. So far as my experience with the disease is concerned I have had many successes and some failures, but I feel very much encouraged. We know very little about the etiology of this trouble, and there is an extensive field that lies before the young men of our profession along this line.

So far as bacterial conditions are concerned, bacterial pathology is a subject which should stimulate our young men to investigate and to make some experiments, in order that we may come to a conclusion as to the cause of the different varieties of pyorrhea alveolaris. It is evident to my mind that there are varieties of the disease which produce the same results, a destruction of both hard and soft tissues.

Coming to the treatment, so far as my experience goes, it is a surgical disease; and it requires a knowledge of the parts and the skillful use of proper instruments to remove the deposits that may be in the pockets or about the teeth; in other words, we should make a clean surgical wound of the pocket and surroundings, being thoroughly aseptic in our treatment, and invite Nature to bring about the formation of new tissue. Whatever that tissue may be I am not prepared to say. But I know from results which I have seen that such tissue is formed, the teeth grow firm, the surrounding parts become healthy, and the patient is made comfortable. Perfect rest, cleanliness and stimulation of the parts must be maintained, or there will be more or less trouble. The case will require careful looking after.

As to the uric acid diathesis, I have read with a great deal of interest what some of our Eastern practitioners have had to say in reference to this matter; and while Dr. Black says that there may be some uric acid deposits in these pockets, I do not think such deposits are the sole cause of pyorrhea alveolaris. I had one case which, after treatment, I dismissed about a year ago. It was one of the worst cases of rheumatic gout that I have ever seen, where the articulation of the thumb- and finger-joints was almost destroyed. Under the use of lithia waters the patient has been made comparatively comfortable; having lost no teeth, and having no decayed teeth. This was a case of pyorrhea alveolaris which extended over the entire mouth, but which involved more particularly the molars. On examination of the case the firsi time, I found the gums everted and thickened. I could pass a probe almost to the apex of the roots of the molar teeth. I saw the patient the other day, and outside of some ordinary calcific deposits on the lower front teeth, I found no pyorrhea. I took a fine probe, and passing it around the necks of the teeth I found the pockets closed. To-day there exists simply a dimple in the gums where the pockets were.

With regard to systemic conditions in these cases, I invariably consult the family physician; and I find in females with female troubles, especially where there is leucorrhea, that unless this condition is controlled we shall fail to cure the pyorrhea alveolaris. The systemic causes must be overcome, and the dentist must act in conjunction with the family physician. Take a large percentage of the cases where there are several pockets, and by thorough instrumentation and the use of lactic acid to produce adhesive stimulation, being careful to maintain thorough asepsis in your operations from start to finish, you can effect a cure in most of them. One of the essential points in the treatment of pyorrhea alveoloris, as in any surgical operation, is to be absolutely clean. Your instruments should be kept in a solution of sulpho-naphthol during the operation, and thorough cleanliness should be maintained by washes after treatment. Take many of the cases that come to us from the country—people who do not care as well for their mouths as those who live in cities. It is my invariable custom to resort to a system of massage; that is, where the gums are swollen and tumefied. After getting through with the surgical part of my work, I pinch these gums as hard as I can to relieve engorgement before making the final applications. Having done this, the pockets can be sealed up by the use of lactic acid. If, on re-examination of cases treated, I find a pocket not closed, I conclude that there must be some remaining deposits and I proceed to remove them with instruments, or a 5 per cent. solution of trichloracetic acid, as suggested by Dr. Harlan, which will act as a solvent in such cases. Having removed the deposits and all dead tissue, soft or hard, close up the cavities and give Nature a chance to do the rest of the work.

I followed Dr. Harlan's advice with regard to treatment of recession of the gums with iodide of zinc, and it has surprised me what success I have had. By making vertical and horizontal incisions and cauterizing the gum with iodide of zinc, the "V-space" can be reduced one-half at least.

I do not rely upon lactic acid to remove deposits; but upon Dr. Younger's improved forms of instruments, to be used by me in my clinic before this society in demonstration of his successful treatment of pyorrhea alveolaris.

Dr. Thomas L. Gilmer, Chicago: I have been very much interested in Dr. Wassall's paper. He has covered practically all the points regarding our knowledge of this disease. I wish to relate in this connection an incident: About twenty-six years ago, Dr. Chase, of St. Louis, was the professor of operative dentistry at the Missouri Dental

College, where I was attending lectures. At that time this disease was not understood or discussed as scientifically and thoroughly as it now is. It was more frequently spoken of as tumefied gums. Dr. Chase was a homœopath. He told the students that if we would use very small doses of calomel, or as he called it, mercurius vivus, we would get beneficial results in the treatment of this disease. I afterward used it to some extent and did seem to get benefit from it, but finally discarded it. About a year ago one of my patients who had pyorrhea alveolaris, disappeared. Seemingly I could only alleviate the condition in her case by the ordinary treatment. She is a sort of semi-invalid, and is frequently in the hands of one of the most eminent physicians in Chicago. Two or three months ago she returned. I supposed, unless she had had treatment by some one else, that her teeth would be loose or lost, but was surprised to see the condition of her gums when she assured me that she had seen no other dentist. The diseased condition had largely disappeared. There were some serumal deposits; still the gums looked healthy and were hugging up around the teeth closely. I asked her what she had been doing for her teeth, and she replied, nothing, but that her doctor had treated her for some other ailment on very minute doses of calomel. This she had taken for several months. Of course, I do not know that the calomel had anything to do with the cure of her gums. I saw the condition her teeth were in then and the condition they are in now.

Dr. Cushing: Was this homœopathic calomel or ordinary calomel?

Dr. Gilmer: I believe she was receiving one-twentieth of a grain of the calomel treatment daily. Of course physicians recognize now that three pellets of the twentieth of a grain of calomel is ample to stimulate the liver.

Since so much has been said about the rheumatic diathesis being the cause of pyorrhea alveolaris, I have watched my patients who have had this disease very closely, and it is the rarest thing for them to acknowledge ever having had rheumatism. On the other hand, I have found that rheumatic patients generally have a good deal of erosion of the teeth, particularly of the occlusal surfaces.

I think Dr. Lawrence is correct in his statement that it requires surgical treatment in order to effect a cure in pyorrhea alveolaris. If we immobilize the loose teeth and remove all the deposits, and all the carious portion of the alveolar border, we are very likely, without any other treatment, excepting perhaps the use of a detergent, to effect a cure. I have in some instances omitted stimulants in the treatment of the disease, relying on surgical procedure and thorough irrigation, and find I get as good results as when I have used stimulants. Surgeons often, by curetting the walls of a cyst, stimulate new and healthy tissue and cause a cure. We do the same in the treatment of pyorrhea. I think small flame-shaped burs are very useful in removing deposits.

Dr. J. G. Reid, Chicago: I know very little about this subject, and wish simply to relate an incident which Dr. Gilmer has touched upon, and which makes me think that this is one of the most difficult troubles we have to contend with. We listen and think, and it sems to me it is nothing but think, think, think, and when we have gotten through thinking, the case is done with. The case I have to relate is one that has given me more trouble than any other case I ever had. It was similar to the one related by Dr. Gilmer. A man had pyorrhea alveolars; his hair was falling out; his teeth were getting loose. He was a young man, comparatively speaking, but he had lost, through my scrupulous care, two or three molars, and he became so debilitated that he had to quit business and put himself under the care of a physician—a homœopathic physician. I saw the gentleman two years later, and expected to see him with his teeth missing, but when he came into my office the teeth that were left were in a healthy condition. This was one of the most aggravated cases of pyorrhœa alveolaris that I have ever come in contact with. I asked the patient if he could relate to me the treatment he had undergone. He coulk not tell anything about it. He said he had faithfully and conscientiously followed the advice of his old homœopathic physician; that the physician kept his pockets full of small pills, which were to be taken as advised. This he had done for two years. When I saw the man's mouth at the end of this time, there was absolutely no indication of pyorrhea in any part of his mouth, and his teeth had not been touched by a dentist in the meantime.

Dr. Cushing: Did you consult the physician regarding the matter?

Dr. Reid: I consulted the physician, but he would not tell me anything about it. He told me I would have to study medicine and find out what he had. I cannot account for the cure of the pyorrhea in this case. I do not know whether it was the physician or not that brought about this result. The man was reasonably clean about his mouth and took the best care of his teeth he could. It seems to me remarkable that such a condition was brought about in this case. I have no theories to offer as to the etiology of pyorrhea alveolaris. We find this condition existing in rheumatic and gouty people, and the gouty and rheumatic people who do not have it are just as numerous as those who do have it, and personally I cannot advance any definite opinion as to the cause of this disease, and I can only say that I do not think we know very much about it at present. Some cases we can and do cure, and there are a great many cases in which we do not effect a cure. I have been tempted oftentimes to experiment a little in some apparently hopeless cases by removing, scraping and replanting them.

Dr. Griffin Munroe, Springfield: In my practice of twelve years I have met with a number of cases of pyorrhea alveolaris, but I very rarely make any promises to my patients as to what I can do for them. Dr. Wassall has suggested that patients with this disease ought to call on the dentist every three months, and I believe this is one of the keynotes to successful treatment of these cases. I have been discouraged to find that some of my patients did not come back at the end of three months for additional treatment, if necessary. I have found one case that relates especially to this trouble which I desire to recite. A gentleman, who lives in Springfield and conducts a business there, thought he had struck a cure for this trouble, so much so that he wanted me to tell it to other people, and it is along the line of homœopathy. He had traveled all the way from Montreal to California, visited dentist after dentist, and when he asked the dentists whether they could do anything for his trouble, they would shake their heads and say the disease was incurable. He found one man that was willing to treat his teeth, and another man in Bozeman, Mont., who had obtained medicine equivalent to Robinson's remedy, which we have often used for this trouble. He persuaded me to send for that remedy. I had used it quite often without any marked results. However, I secured it to please him. After some visits to different dentists, he came back to me and had me to help him a little. He then told me that he was taking a homœopathic remedy, named calcarea, internally, and that he had been taking it constantly for some time. Under the use of this remedy and the attention that he received at my office and from other dentists he happened to visit in the country, he improved considerably, and thought that calcarea renalis was such a wonderful remedy that it ought to be proclaimed to the world.

Dr. Garrett Newkirk, Chicago: There are two or three points I wish to touch upon in connection with this subject. We have heard a good deal about the etiology of this disease to-day, and we know not very much more than we did before, although the paper is an excellent one and presents the facts up to date. A good deal has been said with regard to the removal of the deposits from the roots of teeth. Very well and good, but we must not forget that after the disease has reached a certain stage and there are present large pockets, we have not only to remove deposits from the teeth, but we have to contend with carious bone, real caries of the alveolar process. Dr. Gilmer has touched upon the subject when he spoke of burs for scraping, but I wish to bring it out clearly and emphatically. It is necessary to examine the condition of the alveolar process in these cases, and find out whether or not there are any spiculæ of bone extending into the pockets, because there is often an irregular, ragged condition of these walls. We will find by using a scraper, or a spoon-shaped excavator of the large sort, or a bur, with or without the engine hand-piece, that the walls will break down easily. We should use a bur or other instrument till we feel that we have got down to the harder tissue of the alveolar process. We must get rid of the loose, ragged walls. I have had many cases where, after removing the spiculæ of bone that reached in between the the teeth, the walls being thin, a cure has been effected almost spontaneously with very little other treatment.

A few weeks ago, when I started for California, I gave final treatment in a case of this sort that had been obstinate for a year or more. By means of the bur I reached around in between the roots of the two central incisors and found the softened and easily removable pieces of bone, which I thoroughly took out. Then my assistant during my absence made two or three injections, and when I came home, the case was absolutely cured. The gums were in a perfectly healthy condition. It is just as important to examine the bone as it is to examine the tooth.

One more point: Where you have a case, for instance, involving the lower incisors and possibly the cuspids with them, and there is, perhaps, a crowded overlapping condition of the teeth, extract the worst one of the lot for drainage, then bring the others together with ligatures. You may effect a cure in this manner often when you cannot do it in any other way. If you have a lower incisor or bicuspid which is just hanging by the end of the root, I believe it is better to have it out of the way, to relieve congestion, secure drainage, give room for treatment, and save the rest of them.

Dr. F. H. McIntosh, of Bloomington: I cannot say how intensely interested I am in this subject, and perhaps my method of treatment will help some one, and so I will tell you the method I employ in the treatment of these cases in the way of medicaments. I syringe out the pockets, each of them, with a 5 per cent. solution of pyrozone, and following that I use a 10 to 15 per cent. solution of aromatic sulphuric acid. I get excellent results from this method. Most of you understand the action of aromatic sulphuric acid. It takes hold and removes dead portions of bone, and does not affect the living.

Dr. J. G. Templeton, Pittsburg: Like some of the other gentlemen who have spoken on this subject, I must confess that I know very little about it. I have been treating cases of pyorrhea alveolaris since 1867, at a time before Riggs wrote anything on the subject. At that time I could get nothing to read on the subject, except what was in Taft's "Operative Dentistry" and what was written by the older men of the profession; there was a little in Harris' works, but not very much. When Dr. Robbins, of Marysville, found out what I was doing, he laughed at me. He said he had been all through this thing, and I would have to quit too. I remember saying to him that I did not expect to quit, but that I was going to keep on trying to find something to cure this disease. I asked everybody for information and got nothing. The first I saw was Riggs' work. I did not like it. I had an experience prior to that time not to like what he wrote, when he recommended tincture of myrrh. All I did know was to remove the deposits with suitable instruments as I could get, and I have in my possession now, for removing deposits of tartar, instruments that were made fifty years ago. The man who owned them was killed at the battle of Fredericksburg. I got them from his son and I have used them up to this day. He had been practicing some twenty-five years before he entered the army. As Dr. Reid said, we do not know very much about pyorrhea alveolaris—at least, I do not, although I have been studying it all this time. I believe I have read almost everything that has been published on the subject, and have received some hints from what Dr. Harlan has written, and some points from what Dr. Pierce has written. I do not place much confidence in the uric acid theory. A few years ago, Dr. Patterson took the ground that pyorrhea was due largely to catarrh. In a person who has catarrh badly, this trouble is likely to be aggravated, just as a uric acid diathesis will aggravate it. The best thing, in my opinion, that has been written on this subject in the way of illustration and description, as has been mentioned here, can be found in the "American System of Dentistry." in the article written by Dr. G. V. Black.

In regard to the treatment of pyorrhea alveolaris: I have practically gone through the materia medica in search of remedies to cure this disease. I have tried everything that has been recommended, and I always get back to one thing. Dr. Peck will recollect that I treated some cases at the Chicago College of Dental Surgery a few years ago, and I have to say this in regard to these cases, that there is a great deal called pyorrhea that is not pyorrhea. Dr. Black makes an accurate distinction between cases when he says that certain cases are nothing more nor less than calcic inflammation.

It is an inflammation of the gums caused by the deposit of tartar around the necks of teeth producing irritation. It is different from genuine pyorrhea. Pyorrhea means a flow of pus from the sockets, from the alveolus, and when you see a genuine case of pyorrhea alveolaris, you do not see much deposit. The pus flow is dissolved, it is not oozing out, and there may be some deposits upon the roots of the teeth. It has been said by Dr Lawrence, and very properly, that we may remove all the deposits. I think that is the first thing we should do. If you use the proper instrument and have an educated touch to your finger, you will know when it comes in contact with any of these deposits. Sometimes it requires considerable force to dislodge them.

A few words more in regard to treatment and I have done. I was invited to read a paper before this society, and have prepared one on practical things in practice. At the time I did not know there was to be a paper read on this subject. I have prepared a few lines in regard to pyorrhea alveolaris, and I shall be glad to tell you something about it to-morrow when I read the paper. I will simply say that my sheet-anchor in the treatment of pyorrhea alveolaris is sulphate of copper. Dr. McIntosh referred to the use of aromatic sulphuric acid; that comes nearer to my treatment than anything that has been mentioned this afternoon. You all know that where there is necrosis of bone the sulphuric acid treatment is the most reliable, and that you have to depend upon it to arrest necrosis. I applied it heroically in many cases. I learned to use it at the Pennsylvania Hospital in Philadelphia. While there I saw many cases brought into the clinic presenting large ulcers on the limbs and different parts of the body. These ulcers were indolent and would not heal. We applied a 20 per cent solution of sulphate of copper on a cloth saturated with it, which was laid over the ulcer, and the next time we saw the patient we could see that the ulcer was beginning to heal, that granulations were taking place. At the end of a few weeks the ulcer was entirely healed. After seeing it used in the Pennsylvania Hospital for the treatment of ulcers, I thought of these spongy gums that I had been trying to cure. When I reached home, in the first case that came to my office I thought of this treatment of 20 per cent. solution of sulphate of copper. I applied it and had excellent results.

Dr. Wassall: There is very little for me to say in closing the discussion on my paper. There does not seem to have been any criticism upon it. The discussion, however, has been so instructive and interesting that I think those of us who heard it have been benefited, and I am glad I selected the subject of "Pyorrhea Alveolaris" for my paper.—*Dental Review.*

---

ARTICLE II.

### SOME POINTS IN THE ETHER-CHLOROFORM CONTROVERSY.

